# Spectral Unmixing: Analysis of Performance in the Olfactory Bulb *In Vivo*

**DOI:** 10.1371/journal.pone.0004418

**Published:** 2009-02-09

**Authors:** Mathieu Ducros, Laurent Moreaux, Jonathan Bradley, Pascale Tiret, Oliver Griesbeck, Serge Charpak

**Affiliations:** 1 INSERM U603, Paris, France; 2 CNRS UMR 8154, Paris, France; 3 University Paris Descartes, Paris, France; 4 CNRS UMR 8118, Paris, France; 5 Max-Planck-Institut für Neurobiologie, Martinsried, Germany; Vrije Universiteit Amsterdam, Netherlands

## Abstract

**Background:**

The generation of transgenic mice expressing combinations of fluorescent proteins has greatly aided the reporting of activity and identification of specific neuronal populations. Methods capable of separating multiple overlapping fluorescence emission spectra, deep in the living brain, with high sensitivity and temporal resolution are therefore required. Here, we investigate to what extent spectral unmixing addresses these issues.

**Methodology/Principal Findings:**

Using fluorescence resonance energy transfer (FRET)-based reporters, and two-photon laser scanning microscopy with synchronous multichannel detection, we report that spectral unmixing consistently improved FRET signal amplitude, both *in vitro* and *in vivo*. Our approach allows us to detect odor-evoked FRET transients 180–250 µm deep in the brain, the first demonstration of *in vivo* spectral imaging and unmixing of FRET signals at depths greater than a few tens of micrometer. Furthermore, we determine the reporter efficiency threshold for which FRET detection is improved by spectral unmixing.

**Conclusions/Significance:**

Our method allows the detection of small spectral variations in depth in the living brain, which is essential for imaging efficiently transgenic animals expressing combination of multiple fluorescent proteins.

## Introduction

The use of fluorescent proteins [1] has led to a huge up-turn in the production of functional and anatomic information. In the last decade spectral variants of these proteins have been cloned or engineered [2], and introduced in transgenic animals either to report neuronal activity [3]–[9] or to label neuronal subsets [10] or subtypes [11]. Efficient detection of several fluorescent proteins in the living brain requires sensitive spectral imaging techniques to resolve small spatial differences and temporal changes in color. The expression of multiple fluorescent proteins in single cells or in spatially overlapping cellular processes, as well as the large spectral overlap of fluorescent proteins leading to detector spectral bleed-through, impose specific challenges on any system with the goal of attributing a given color to a specific cell.

Spectral unmixing (SU), in which the sample is imaged onto several spectral channels, specifically addresses the issue of detector bleed-through [12]–[16]. Under the assumption that the spectrum of an individual fluorophore is linearly summated on each image pixel, SU extracts the weight (or contribution) of each individual spectrum, with that weight proportional to the fluorophore concentration [14]. SU has been implemented in two fashions, sequential [17]–[20] or parallel spectral imaging [12], [21]. Sequential spectral scanning uses tunable bandpass filters [17], [18], liquid-crystal filters [19] or acousto-optic filters [20]. Parallel spectral imaging, on the other hand, samples fluorescence simultaneously onto several detectors allowing fast image acquisitions, a prerequisite to follow fluorescence transients. To date the latter has only been used in the confocal mode [22]–[25] and thereby suffers limitations when imaging in depth in brain tissue [26], [27].

For the present study, we built a simple and light-efficient spectral imaging apparatus using a two-photon laser-scanning microscope equipped with a parallel 4-channel detection system. With this we investigated the advantages and limitations of SU over conventional dual channel ratiometric methods using two well characterized FRET-based reporters of calcium (Yellow Cameleon (YC3.1) and CerTN-L15). Both theses reporters are genetically encoded fusions of calcium binding moieties (calmodulin and troponin-C, respectively) and two fluorescent proteins having overlapping spectra, and undergo a change in FRET efficiency in response to changes in intracellular [Ca^2+^]. First, we assessed the performance of our method in shot noise limited conditions. Then, we assayed SU for the detection of calcium dependent FRET signals from the calcium reporter YC3.1, which was co-expressed with ATP-gated P2X_2_ calcium channels in human embryonic kidney (HEK) cells and stimulated with ATP. In transgenic mice expressing CerTN-L15, we investigated the performance of SU on FRET signals evoked in mitral cells of the olfactory bulb in response to odor stimulation. We found that with SU, FRET sensitivity was improved, and that reporter efficiency was limiting. Computer simulations supported these conclusions.

## Materials and Methods

### Setup

**Figure 1:**
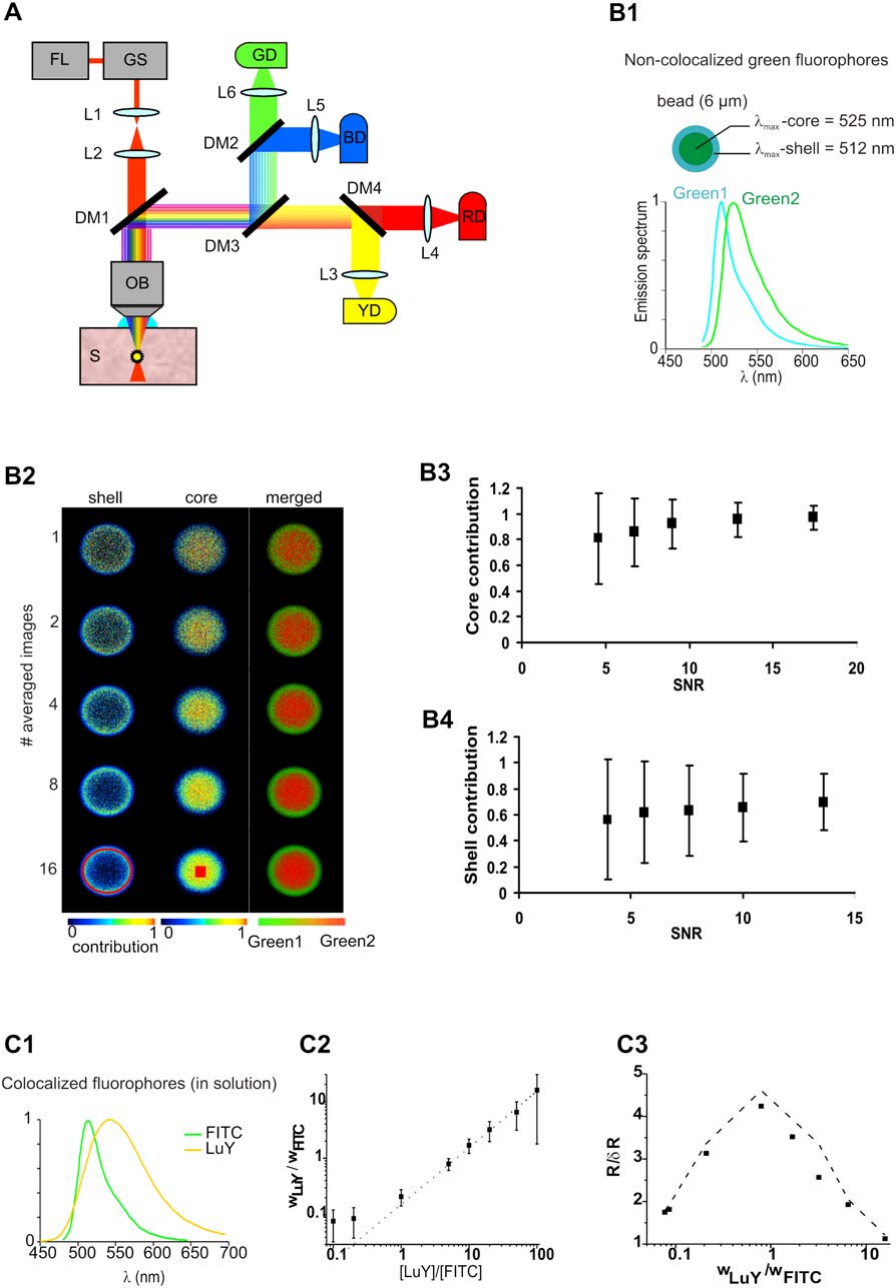
Two-photon excitation fluorescence (TPEF) and spectral unmixing (SU) of calibration samples. (A) Experimental set-up combining TPEF and 4-channel spectral detection. For more details and abbreviations see experimental procedures and Table S1. (B) SU of 2 non-co-localized fluorophores with highly overlapping emission spectra. (B1) Sample (beads) spectral properties. The shell and core emission spectra are separated by only 13 nm. (B2) Core and shell contributions and merged abundances as a function of averaging. Separate and merged contribution images of shell and core fluorophores in the equatorial plane of a bead. Improving the signal to noise ratio (SNR) by averaging sharpens the limit between the shell and core. B3-4) Plots of shell and core contributions versus SNR measured in red ROIs (same number of pixels) in bottom row of B2. Contributions rise asymptotically and noise decreases with SNR (error bars: +/−SD) Note that the shell SNR is smaller than that of the core because the fluorescence emitted at the edge of the bead is smaller. C) SU of co-localized fluorophores (fluorescein isothiocyanate (FITC) and lucifer yellow (LuY)) at different concentrations in solution. (C1) Emission spectra of FITC and LuY. C2) Ratio of the two measured fluorophore contributions as a function of their concentration ratio. The proportionality indicates that measuring the contribution ratio allows determination of the relative concentration of the fluorophores. (C3) Estimation of the contribution's ratio relative precision. Plots of the experimental points (black diamonds) and simulation curve (dashed line). The ratio relative precision (R/δR = (σw_LuY_/w_LuY_ + σw_FITC_/w_FITC_)^−1^) is maximum when the contribution ratio is close to 1.

We used a custom-build two-photon laser scanning microscope (Figure 1.A) equipped with a femtosecond Ti:Saphire laser (FL) (Tsunami^TM^, Spectra Physics, Mountain View, CA, USA), tunable from 740 to 1000 nm, and galvanometric scanners (GS) (VM500, General Scanning, Watertown, MA, USA). Pixel dwell time was 6 µs for all images. A telescope (L1–L2) was used to expend the beam 3 times. All images were acquired with a 63x water immersion objective (HCX APO, 63×, NA 0.9, Leica, Germany). Collected fluorescence was split into four spectral channels composed of three dichroic mirrors DM2, DM3 and DM4 and four photomultiplier tubes (PMT) (R6357, Hamamatsu, Japan): BD, GD, YD and RD. Emission filters could be placed in front of each detector (not displayed in Figure 1.A). Low resolution, four point spectra were acquired in parallel for all pixels. The set of dichroic mirrors and emission filters used for each experiment presented in this paper are specified in Table S1. The selection of dichroic mirrors was based on the method described in [28]. We computed the three cut-off wavelengths that maximized the sum of square SNR of each contribution. Cut-off wavelengths were moved in 5 nm steps and all possible combinations of three cut-offs were tested. We then selected standard off-the-shelf dichroic mirrors with cut-off as close as possible to the optimization results. Computed channel spectral responses are displayed in Figure S1 for unmixing green1/green2 dyes or LuY/FITC, and Figure S2 for unmixing ECFP/EYFP. The four PMTs internal gains were set to identical levels. PMT signals were amplified by a custom-built electronic circuit and digitized by an A/D board (PCI-6110, National Instruments, Austin, TX, USA). The amplification gains were automatically recorded and used for normalization of spectral images. Image acquisition, display and analysis were controlled with a program written with LabVIEW (National Instruments, Austin, TX, USA).

A practical point to be noted is that the cut-off wavelength of dichroic filters depends slightly on the angle of the incident light, as a consequence the spectral sampling varied as a function of the position in the image. We estimated that in our case the dichroic cut-off wavelengths shifted by about +/−6 nm over the total lateral field of view (320 µm, 63× objective). This small effect could produce unmixing bias, so to circumvent this problem we acquired reference spectra and analyzed signals only in the central part of the images (100X100 µm^2^ area). We are currently investigating a corrective method to allow accurate SU over the entire field of view.

### Spectral unmixing

We used a maximum likelihood (ML) analysis to spectrally unmix multiple fluorophores. For a description of the ML analysis applied to SU see [29]. The solution of ML analysis was a set of fractions computed with a precision of 0.005 for each fluorophore present in the sample. The contributions were obtained by multiplying the fractions by the measured light intensity. We compared ML algorithm with the standard least square algorithm commonly employed to solve linear unmixing problems [14] by simulating four channel spectral detection of fluorescence from CFP/YFP solutions with variable intensities. We found that ML provided the highest precision of contribution ratio, in particular for small photon counts (data not shown). Davies and Shen also observed advantages of using the ML analysis for SU [29].

### Fluorescent microsphere standards

We imaged 6 µm diameter double-green fluorescent microspheres (FocalCheck^TM^, Molecular Probes®-Invitrogen, Carlsbad CA, USA) with shell and core overlapping emission spectra (shell-green1 = 512 nm, core-green2 = 525 nm) (Figure 1.B1). Laser excitation wavelength was 850 nm. We selected dichroic mirrors DM2, DM3 and DM4 with cut-off wavelengths at 510, 530 and 570 nm respectively. The sensitivity in the four channels is displayed in Figure S1.A. Bead images were acquired in the equatorial plane. Green 1 and green2 contributions were measured in an annulus within the shell and in a small square in the center of the bead respectively. Both regions of interest (ROIs) contained 300 pixels. The signal to noise ratio (SNR) measured in these ROIs was defined as the mean intensity divided by the standard deviation.

### LuY/FITC solutions

100 µM aqueous solutions of FITC and Lucifer Yellow (LuY) at pH = 8 (buffered in HEPES) were mixed to obtain [LuY]/[FITC] ratios between 0.1 and 100. FITC and LuY two-photon excitation cross-sections are well characterized [30], [31]. Small glass wells were filled with the solution and covered with a coverslip. A 30×30 µm^2^ (∼30×30 pixels) region was imaged at 860 nm excitation wavelength with ∼2 mW laser power. LuY and FITC were unmixed and the average and standard deviations of the ratio of contributions w_LuY_/w_FITC_ were measured for each [LuY]/[FITC] solutions. Emission spectra of LuY and FITC peak at 513 nm and 544 nm respectively, and present a strong overlap. We used dichroic filters with cutoff at 510 nm, 530 nm, and 570 nm. The four channel spectral sensitivities are presented in Figure S1.B. The simulated contribution ratio precision was generated with the same algorithm as the simulations of FRET signal detection (see below). The four channel spectra of FITC and LuY used as input parameters were measured in pure samples. The number of photons was set as the square of the intensity image SNR (shot noise limited conditions). Input fractions of LuY and FITC for each simulated sample were determined by the ratio of concentrations multiplied by the two-photon action cross sections.

### HEK cells co-transfected with YC3.1 and P2X_2_

#### Experimental protocol

HEK cells plated on a polylysine-coated glass coverslip were co-transfected with YC3.1 and P2X_2_ DNA plasmids using Exgen 500 (Euromedex, France). 24–48 hrs after transfection, cells were placed in a recording chamber under the microscope and perfused with extracellular control solution (concentrations in mM: NaCl (140), KCl (5), HEPES (20), Glucose (25), CaCl2 (2), MgCl2 (1) at PH 7.4). Cells were stimulated every 10 min with a 30 s application of 50 µM ATP. This cellular paradigm and stimulation protocol provided a reproducible and robust model that allowed a reliable comparison of three methods (BP, DC and SU) to detect FRET activity (see below). The excitation wavelength was 800 nm and power at the sample was ∼2 mW. Two photon excitation cross section spectra of ECFP and EYFP were measured by Zipfel et al. [32]. At 800 nm, the ratio EYFP over ECFP contributions in control conditions were close to 1 providing maximal precision (see Results). To study the effect of biological tissue light scattering and absorption on FRET detection, a 300 µm thick brain slice of rat neocortex was placed above the cells. The glass coverslip was flipped upside-down so that ATP could directly reach the cells. The average laser power incident on the sample varied between 100 mW and 220 mW.

### Image analysis and FRET indices

All images were background corrected and low-pass filtered by convolution with a 3x3 pixel gaussian kernel. Background was measured in an ROI devoid of fluorescent cells. ECFP and EYFP reference spectra, used for SU, were acquired in HEK cells expressing pure ECFP or EYFP. We also verified that the low resolution four-point spectra of Cerulean (Citrine) and ECFP (EYFP) are identical (data not shown). We compared three FRET detection methods: (1) the classical bandpass BP method (535/475), (2) a two-channel dichroic DC method (>510/<510), and (3) four-channels SU. To achieve the BP method, we removed dichroics DM2 and DM4 and placed emission filters BP460-490 (Olympus, Tokyo, Japan) and 535DF35 (Omega Optical, Bratelboro, VT, USA) in front of detectors GD and RD. The FRET ratio of the DC method was the sum of YD and RD detector signals, divided by the sum of BD and GD signals, i.e. approximately the ratio of intensities above and below 510 nm.

For each cell, the response to ATP stimulation was recorded successively with two channels to measure R_BP_, and with four channels to record both R_DC_ and R_SU_. FRET indices were averaged over the entire cell cytoplasm. Relative ratio variations (in percentage) were defined as
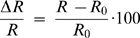
(1)where R_0_ was averaged over a 40s pre-stimulus period. Although R_0_ can vary between measures, most researchers use ΔR/R and therefore we used this parameter in order to compare our results with published data.

We also used the sensitivity ΔR/σR, defined as the largest ratio increase (R_max_−R_0_) divided by σR, the standard deviation of the ratio measured in a 40 s control period. Sensitivity characterizes the ability to detect stimulation-specific FRET responses over pre-stimulus fluctuations. Furthermore, since sensitivity is not defined relative to R_0_, it is a more appropriate criterion to compare methods.

### In vivo imaging of FRET-based FCIP

All animal experiments were performed in accordance with INSERM guidelines. We used transgenic mice expressing the troponin-C-based Ca^2+^ sensor CerTN-L15 under the control of Thy1 promoter [8]. Animal preparation and surgery were performed as in [33]. We imaged a field of view containing 1–8 mitral cells at depth between 180 µm and 250 µm with frame rates of 3–6 Hz. Animals were stimulated for 3 seconds with various odors (benzaldehyde, iso-amyl acetate, ethylpropionate) at saturated vapor pressure. Stimulations were repeated between 3–10 times (interstimulus interval = 2 mn). Laser wavelength was 800 nm and power varied between 30 mW and 100 mW. Responses were averaged (n = 3 to 10). Cells were considered non-responsive for ΔR/σR<1.8.

### Computer simulations of FRET signal detection

We designed a model with two fluorophores (e.g. EYFP and ECFP) and 4 channels that could easily be generalized to n fluorophores and m detectors, provided n< = m. Assuming N_ph_ photons were emitted by a sample containing ECFP and EYFP, for which the normalized reference spectra (S_i,j_) were measured experimentally; assuming f is the relative contribution of ECFP (and 1−f for EYFP); the distribution of photons between the 4 channels is computed by a multinomial distribution where the probability that a photon is detected by detector j is given by p_j_(f) = fS_1,j_+(1−f)S_2,j_. We used a Monte-Carlo algorithm to generate the detectors photon counts (n_1_, n_2_, n_3_, n_4_), with Σn_i_ = N_ph_. Then the ML algorithm determined f, the measured ECFP fraction that maximize the likelihood of the distribution (n_1_, n_2_, n_3_, n_4_) knowing S_i,j_. Finally the FRET ratios R_DC_ and R_SU_ were defined as:
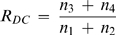
(2)

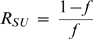
(3)We computed the temporal average and standard deviation of R_DC_ and R_SU_ by repeating this process Nt times. FRET changes simulating FCIP responses were generated by changing f from f_control_( = 0.5) to f_response_ corresponding to ΔR/R_SU_ = 25, 50, 75, 100, 125, 150, 175, 200%. Sensitivities ΔR/σR for R_DC_ and R_SU_ were finally computed as well as the gain in sensitivity between SU and DC.

The following input parameters were used:

S_ECFP,j_ = [0.236, 0.356, 0.217, 0.19]

S_EYFP,j_ = [0.0294, 0.0966, 0.429, 0.445]

N_ph_ = 10^2^, 10^3^, 10^4^ photons

N_t_ = 50,000 time points

S_ECFP,j_ and S_EYFP,j_ were measured experimentally in HEK cells transfected with ECFP or EYFP DNA plasmids.

## Results

### Characterization of SU performances using fluorescent standards (shot-noise limited conditions)

We performed SU using the microscope described in Methods and Figure 1.A.

To test our method in shot-noise limited conditions we imaged double-green fluorescent microspheres in which the shell and core dyes had fluorescence emission maxima separated by only 13 nm (Figure 1.B1). By averaging an increasing number of images, and unmixing the contributions of each dye (Figure 1.B2), we found the relative contribution of the core increased from 0.81 to 0.97 as the signal to noise ratio (SNR) improved from 4.6 to 17.4 (Figure 1.B3). Simultaneously, the uncertainty decreased by ∼4 fold. The same trend was observed for the shell, whose contribution increased from 0.56 to 0.70 as the SNR improved from 3.9 to 13.6 (Figure 1.B4). Since the lateral resolution of our microscope (0.6 µm) exceeded the shell thickness, a small amount of core dye was present in the shell ROI. As a result, the shell contribution did not tend to 1. Averaging also improved the spatial separation between the two dyes as shown in the merged images (Figure 1.B2, right column). This first result demonstrates that 4 channels are sufficient to spectrally unmix two fluorophores with strongly overlapping spectra, provided the SNR is roughly greater than 10.

We then evaluated SU with respect to spectrally overlapping co-localized fluorophores, a situation that mimics a FRET-based fluorescent calcium indicator protein (FCIP). For three reasons we used FITC and lucifer yellow (LuY) in solution as fluorophores: 1) because the relative brightness of LuY and FITC can be easily adjusted by changing [LuY]/[FITC] in solution, 2) because they cannot be efficiently separated without SU (Figure 1.C1), and 3) because their two-photon action cross-sections are known. The mean ratio of the LuY to FITC contribution (w_LuY_/w_FITC_), when expressed as a function of the dye concentration ratio ([LuY]/[FITC]) ranging from 0.1 to 100, was close to the expected linear dependence (Figure 1.C2). Indeed, assuming linear summation of fluorophore intensity, the measured contributions were proportional to the fluorophore concentration and two-photon action cross section. The slope of the linear fit (0.153) was similar to that obtained from published data (0.148) [30], [31], [34]. For a given SNR (∼25), the relative precision R/δR (R = w_LuY_/w_FITC_, δR = variability of R) was maximal for w_LuY_/w_FITC_ close to unity (Figure 1.C3). A model simulating spectral fluctuations, based on the experimental fluorophore reference spectra and detected mean intensities (see methods), confirmed our measurements (Figure 1.C3, dashed line). The good accordance between computed and measured R/δR shows that statistical fluctuations of photon count ultimately limits the precision of R. This result also indicates that the FRET ratio precision is maximal when the donor and acceptor contribute equally to the fluorescence signal.

### SU improves detection of FRET-based FCIPs signals in vitro

**Figure 2:**
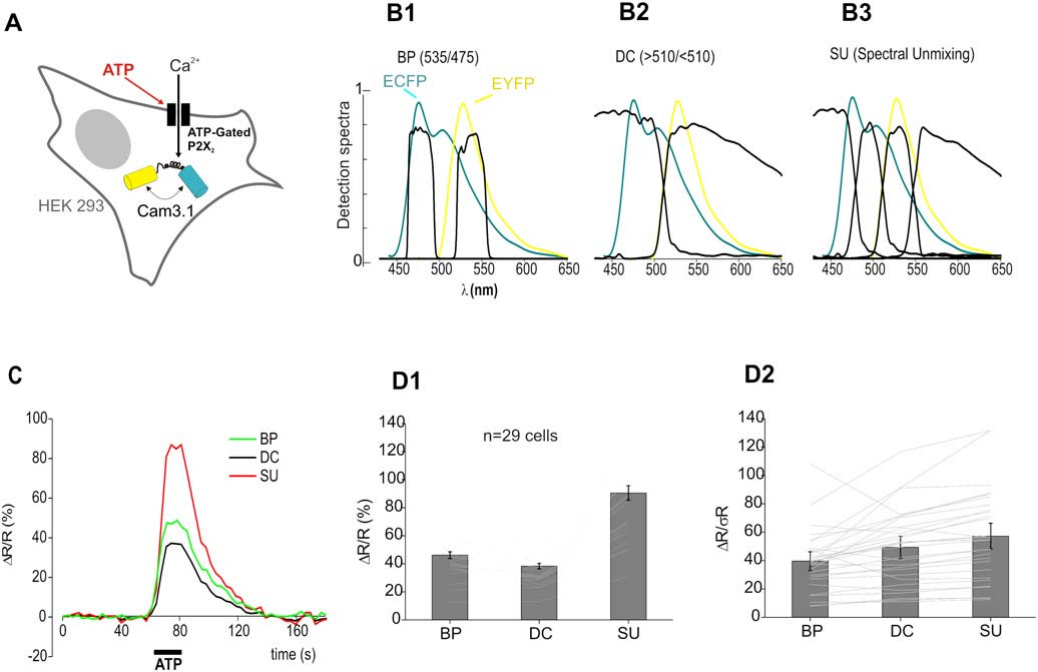
Improvement of FRET signal detection with SU. (A) Model cells: HEK293 cells expressing YC3.1 and P2X_2_ receptor channels. ATP induces an intracellular Ca^2+^ rise that reliably triggers a YC3.1 FRET signal. (B1–B3) Comparison of the detector spectral efficiencies used in 3 methods to detect FRET: 535/475 (BP), (λ>510 nm)/(λ<510 nm) (DC) and spectral unmixing (SU). Emission spectra of ECFP (blue) and EYFP (yellow). (C) SU improves the ATP normalized FRET response compared to BP and DC methods (green: BP; black: DC; red, SU). Single cell, 30 s bath application of 50 µM ATP, signal integrated over the entire cell cytoplasm. (D) Summary graph of all responses (n = 29 cells). (D1) SU improves FRET responses. (D2) plot of the measurement sensitivity in the three conditions. σ_R_: standard deviation of the ratio before ATP application. ΔR: FRET response.

**Figure 3 pone-0004418-g003:**
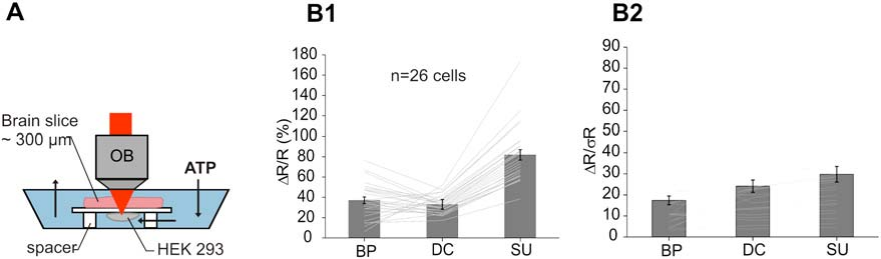
Improvement of FRET signal detection in depth *in vitro*. (A) schematic of the experimental set-up used to record FRET signals in depth from HEK293 cells expressing YC3.1 and P2X_2_ receptor channels. The HEK cell culture dish was placed upside down and below a 300 µm thick neocortical mouse brain slice. ATP was bath-applied. (B1) SU improves FRET responses compared to BP and DC methods. (B2) plot of the measurement sensitivity in the three conditions. σ_R_: standard deviation of the ratio before ATP application. ΔR: FRET response.

We then tested how our method performned in detecting FRET-based FCIP signals using HEK 293 cells co-transfected with YC3.1 and P2X_2_ plasmids. Bath application of ATP (50 µM) opened P2X_2_ channels (Figure 2.A) triggering a rise of the intracellular [Ca^2+^] that increased the FRET signal. Three FRET indices were measured (Figure 2.B and methods): (1) the classical bandpass 535/475 ratio (R_BP_), (2) a two-channel dichroic ratio (R_DC_, cut off = 510 nm) and (3) the four-channel SU ratio of EYFP and ECFP contributions (R_SU_). In these cell cultures, images were acquired in shot-noise limited conditions. Figure 2.C illustrates the response of a single cell to ATP and shows that SU significantly improved ΔR/R. Overall (n = 29 cells), ΔR_SU_/R_SU_ (90.5+/−5.2%) was about twice that of ΔR_BP_/R_BP_ (46.2+/−2.4%) or ΔR_DC_/R_DC_ (38.4+/−1.9%), the poor performance of the latter likely resulted from the large bleed through of ECFP into the EYFP (λ>510 nm) channel (Figure 2.D1). We also compared the sensitivity (ΔR/σR) of the three measurement methods, with σR corresponding to the standard deviation of the FRET ratio before stimulation and found that ΔR_SU_/σR_SU_ (57.3+/−5.9) was still better than ΔR_BP_/σR_BP_ (39.6+/−3.9) or ΔR_DC_/σR_DC_ (49.4+/−4.8) (Figure 2.D2). Note that the sensitivity gain due to SU was not as large as the ΔR/R improvement because ΔR/R is normalized by baseline R mean, while sensitivity is defined with respect to baseline noise. With the DC method the increased sensitivity compared to the BP method was due to the integration of more light. Overall, SU significantly improved the sensitivity of FRET detection in comparison to the other methods (p<0.005, paired student t-test). Next, by covering the transfected cells (n = 26) with an acute brain slice (∼300 µm thick, mouse neocortex) (Figure 3.A), we asked how light scattering affects SU. Excitation and collection of fluorescence through the brain slice slightly reduced (∼10%) the amplitude of all three ATP-evoked ΔR/R: ΔR_SU_/R_SU_ = 81.2+/−5.1%, ΔR_BP_/R_BP_ = 37.1+/−3.3%, ΔR_DC_/R_DC_ = 33+/−1.6% (Figure 3.B1). Interestingly, the sensitivity decreased more significantly (∼50%) for all three detection methods: ΔR_SU_/σR_SU_ = 29.8+/−3.7, ΔR_BP_/σR_BP_ = 17.4+/−2.1 and ΔR_DC_/σR_DC_ = 24.1+/−2.9 (Figure 3.B2). Nevertheless, 4-channel SU still provided optimal sensitivity (p<0.005, paired student t-test). Taken together, although light scattering reduces the added improvement in sensitivity of SU to the BP and DC methods, our results suggest that SU should significantly improve FRET signal amplitude and sensitivity *in vivo*.

### SU improves FRET signals in vivo

**Figure 4:**
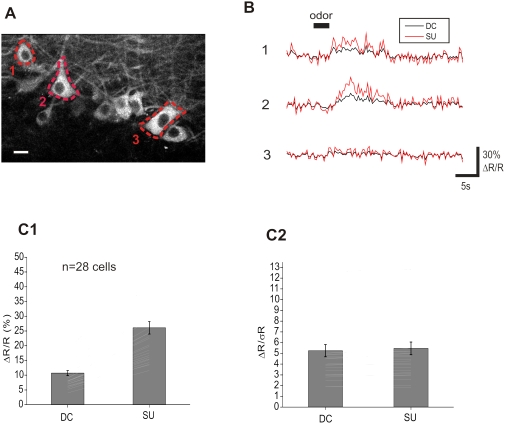
FRET signals and SU in the olfactory bulb of transgenic mice. (A) Image of mitral cells expressing a Troponin C-based Ca^2+^ sensor at 200 µm in depth. Scale bar: 20 µm. (B) Examples of FRET responses to odor (benzaldehyde) stimulation measured in 3 cells delineated in A (average of 5 stimulations). Plots are obtained with either SU (red) or DC (black) methods. Cells that did not respond (e.g. cell 3) were not included in the population analysis. (C) Summary of results on 28 cells. (C1) ΔR/R increases from 10.7+/−0.9 to 26.1+/−2.1% using SU compared to DC method. (C2) Sensitivity (ΔR/σ_R_) is almost identical between SU and DC methods. Error bars: +/−SEM.

In order to compare the 4-channel SU and DC methods of detection *in vivo* we took advantage of transgenic mice that express the troponin-C-based Ca^2+^ sensor CerTN-L15 [8] . Using the DC method, Heim *et al.* have reported a ΔR/R of 50% in superficial cortical dendrites upon iontophoresis of glutamate, *in vivo*. We analyzed odor-evoked FRET responses of mitral cell somata in the olfactory bulb at a depth of 180-250 µm (Figure 4.A). As expected [35], responses were odor- and cell-specific (Figure 4.B). Odor evoked a wide range of ΔR/R changes (SU, 11- 48%; DC, 4- 21%) (Figure 4.C1). Overall (n = 28 cells), ΔR_SU_/R_SU_ was larger (26.1+/−11.2%) than ΔR_DC_/R_DC_ (10.7+/−4.5%), confirming our *in vitro* measurements. On the other hand, the sensitivities measured with both methods were nearly identical: ΔR_SU_/σR_SU_ = 5.5+/−0.6 and ΔR_DC_/σR_DC_ = 5.3+/−0.6 (Figure 4.C2).

How is it that *in vivo*, SU doubled the signal amplitude but had almost no effect on sensitivity, whereas *in vitro*, SU significantly improved both ΔR/R and ΔR/σR? To investigate this discrepancy, we first quantified the gain in sensitivity as:
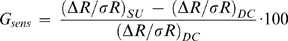
(4)


**Figure 5:**
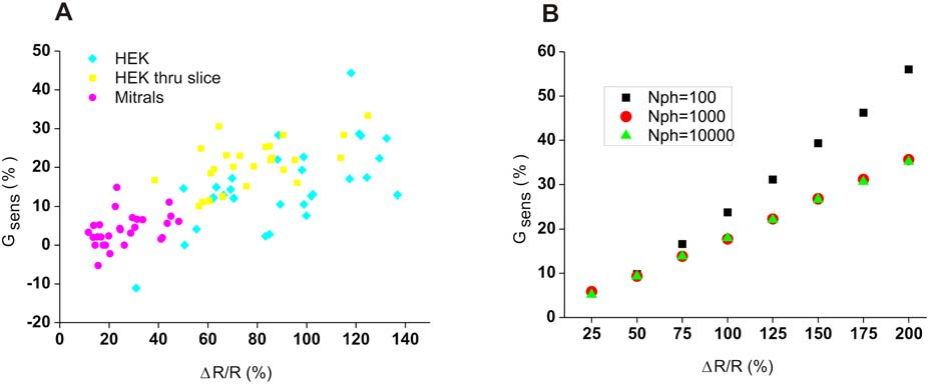
In vivo, SU improvement depends on the FRET probe efficiency. The relative gain in sensitivity G_sens_ strongly depends on the signal amplitude as observed experimentally (A) with HEK cells (cyan diamonds), with HEK cells imaged through a brain slice (yellow squares) and with mitral cells imaged *in vivo* (magenta circles) or theoretically (B). Our simulation also revealed that for small photon counts, G_sens_ further improved, e.g. reaching 55% for N_ph_ = 100 and ΔR/R = 200%.

We then computed G_sens_ for all recorded cells (Figure 5.A) and observed that although G_sens_ was widely distributed on the scatter plot, it clearly increased with ΔR/R. Because the mean ΔR/R for mitral cells (magenta) was small (mean ΔR/R = 26.1%), the average gain was near 5%, which is a moderate improvement. However for the few mitral cells that had large values of ΔR/R, as it was the case for HEK cells *in vitro* (Figure 5.A, yellow squares and cyan diamonds), SU markedly improved the sensitivity. To further investigate this point, we simulated our FRET experimental conditions with a total photon count of 10^2^–10^4^ and a ΔR/R of 25–200%. Statistical spectral fluctuations were generated with a multinomial distribution over the four detectors (see experimental procedures). With this model we found a continuous increase of G_sens_ with ΔR/R (Figure 5.B), thus confirming our experimental data. Interestingly the model also revealed a >20% improvement of G_sens_ for large response amplitudes (ΔR/R>100%) at a low photon count. These results indicate that SU is more efficient than the DC method for the detection of FRET-based FCIP activity, as long as the reporting probe is efficient beyond a certain threshold.

## Discussion

### Spectral sampling

In order to detect the activity of neurons *in vivo* using FRET-based FCIP, or to distinguish labeled cells with strongly overlapping spectra, high resolution of temporal and spatial spectral variations is necessary. Spectral sensitivity is limited by both photon shot-noise in each channel, and by global instrumental noise. We decided to use a four-channel spectral sampling scheme to ensure a high photon count in each channel and reduce the overall instrumental noise. Note that theoretically two channels are sufficient to unmix the donor and acceptor of FRET probes. From numerical simulations we determined that the optimum cut-off wavelength for 2 channel SU of CFP/YFP FRET pairs is 510 nm (data not shown). We did not notice any difference between the results of FRET signal detection with four- or two-channel SU (summing channel 1+2 on one hand and 3+4 on the other hand). The main advantage, therefore of our four-channel detection unit is the added capability of unmixing up to four co-localized fluorophores. Considering read-out noise alone (not shot-noise), Zimmermann et al. previously showed that the efficiency of unmixing was maximal when the number of detectors equaled the number of fluorophores [15]. On the other hand, when photon shot noise dominates, Neher and Neher demonstrated that increasing the number of channels resulted in very little improvement of unmixing efficiency [28]. With our four spectral channels, we obtained a high contrast between two non co-localized fluorophores with spectral peaks as close as 13 nm, a precise unmixing of two co-localized fluorophores in solution with variable concentrations, and finally, unmixing of four fluorescent proteins (ECFP, GFP, EYFP and tdimer2 with peaks at 475, 507, 527 and 579 nm respectively) was achieved (Figure S3). We conclude that four channel SU is sufficient for distinguishing colors with high spectral similarities and to separate up to four fluorophores with overlapping spectra. Taking also into consideration our results with SU in scattering media (Figures 2–4), we believe that it should be feasible to separate multicolor cell populations in the brain with our method. This, for example, would be applicable to imaging from brainbow mice, in which the stochastic expression of three or four fluorescent proteins induces different colors in neurons [36].

### Ratiometric probe imaging

Ratiometric calcium indicators, like FRET based FCIP, have a particular advantage over intensity-based probes in that a ratio between responses at two different emission wavelengths can be used to determine the [Ca^2+^] independently of the collected light intensity [37]. Thus, it is critical that the accuracy and the precision of the measured ratio be optimized. Using variable concentrations of dyes in solution, we demonstrated that the precision of the ratio of unmixed contributions is best for R∼1 (Figure 1.C3). To keep the precision within 20% of its maximum, R should stay between 0.3 and 3. Thaler and Vogel found that the highest accuracy and precision of the measured abundance ratio of a 1∶1 mixture of CFP and YFP is obtained with a two-photon excitation wavelength that creates approximately equal intensity of both fluorophores [38]. They determined that the optimum wavelength is close to the wavelength at which CFP and YFP have the same two-photon action cross-section [32]. In the present work, we decided to use 800 nm light to excite YC3.1 and CerTN-L15. Indeed, at this wavelength, we measured contribution ratios near 1 in control conditions, which ensures the greatest precision of R according to Fig. 1.C3 (1.15+/−0.28 for HEK cells transfected with YC3.1; 0.7+/−0.18 for mitral cells expressing CerTN-L15 probe). This result was somewhat surprising since at this wavelength, EYFP (or Citrine) excitation was expected to be much smaller than ECFP (or Cerulean). The most likely explanation is that, in our experimental conditions, baseline FRET efficiency is sufficiently high to induce a significant transfer of energy from donor to acceptor. Exciting YC3.1 and CerTN-L15 at 800 nm presented another advantage: at this wavelength the ratio of acceptor to donor two-photon excitation cross section is smallest[32], thus creating the largest ΔR/R.

Note that higher wavelengths would excite fluorophores more efficiently producing brighter signals (λ_peak_ = 840 nm for CFP, λ_peak_ = 960 nm for YFP), reducing the noise on R. This might improve FRET signal detection. However ΔR/σR also depends on many criteria such as the resting FRET efficiency, intrinsic Ca^2+^ fluctuations, probe expression level and Ca^2+^ sensitivity. Furthermore, *in vivo,* one should also take into account other considerations such as the increased penetration depth with higher wavelengths or the loss of contrast due to autofluorescence excitation.

To conclude, a more systematic wavelength scan needs to be performed to determine the optimum excitation wavelength for each specific probe and experimental conditions.

### SU in depth

SU of neurons with multiple colors *in vivo* and in depth requires a light efficient spectral imaging method. In contrast to confocal microscopy, which limits detection of fluorescence to few tens microns in depth in the brain [27], [39], our approach allowed us to greatly increase this distance in the anesthetized animal and detect odor-evoked FRET transients up to 180–250 µm in depth in the main olfactory bulb. To our knowledge this is the first demonstration of *in vivo* spectral imaging and unmixing of FRET signals at such depth.

Fundamental issues for measuring depth resolved spectra in thick biological samples are how the tissue optical properties, such as scattering and absorption, vary with wavelength. Light scattering by particles that are small compared to wavelength decreases as 1/λ^4^. For larger particles, scattering tends to oscillate as a function λ. Absorption might also play a role, although to a much smaller extent since the absorption coefficients μ_a_ measured in bulk biological tissues are typically 10 times smaller than the reduced scattering coefficients [40]. As a consequence, the emission spectra of fluorophores inside biological samples, whether measured with our four detectors system or with a finer resolution method, are affected by the nature and thickness of the tissue through which they are recorded. For example, we observed that the fluorescence spectra of HEK cells transfected with YC3.1 was altered when imaged through a brain slice (Figure S4): blue and green channels were attenuated compared to yellow and red channels. The same trend was observed in YFP expressing cells in the cortex of transgenic mice (data not shown). Even though the attenuation amounted to less than 5% of the total intensity, and thus did not affect our results, the tissue optical properties should be systematically considered and tested when performing SU in depth, particularly the spectral dependence of any fluorescent probe. Other factors that could alter the spectrum of a fluorescent cell in depth are the out-of-focus fluorescence, generated either by autofluorescent proteins or by the protein of interest being expressed in overlying cells, and the excitation laser leak that might occur especially when imaging in depth with high powers. In principle, background subtraction cancels out the mean value of these additional signals. However background temporal fluctuations (motion artifact, blood flow, fluorescence shot noise) and spatial non-uniformity will modify the signal intensity and spectrum, and alter the SU. These are some caveats to heed when performing SU in depth. As a consequence, FRET-based FCIP signals measured in depth with SU provide a reliable, yet relative rather than absolute indicator of [Ca^2+^] variations.

### SU for FRET-based FCIPs

We found a clear advantage of using four-channel SU over more conventional methods to detect FRET-based FCIP activity. Our results obtained *in vitro* from HEK cells confirmed previous studies showing improvements of FRET signal detection with SU [41]–[44]. *In vivo*, our results from mitral cells proved more complex: although ΔR/R was still much greater with SU than with the dichroic method, the sensitivity gain was smaller than in the case of the HEK cells (5% instead of 20% on average). What underlies this difference?

First the variation of the FRET index R in the pre-stimulus condition was much greater with mitral cells imaged *in vivo* than in the case of HEK cells (compare Figure 4.B and Figure 2.C) Although this was principally due to the reduced fluorescence intensity detected, it is also possible that other factors including intrinsic [Ca^2+^] variations in mitral cells or dynamic light scattering (e.g. moving red blood cells, blood vessels dilations/contractions) contributed. A second and critical difference between the results *in vivo* and *in vitro* was the larger response amplitude from YC3.1 expressed *in vitro* in HEK cells (∼80%) compared to the CerTN-L15 expressed in mitral cells *in vivo* (∼26%). According to the scatter plot of Figure 5.A, and the simulation results of Figure 5.B, the gain in sensitivity because of using SU would have been larger if a FRET-based calcium probe with a ΔR/R above 100% had been employed. With a small photon count, e.g. when imaging calcium dynamics in sub-cellular domains, or when measuring calcium with a high temporal resolution, the gain form using SU would have been even better. Currently most FRET based FCIPs have reported ΔR/R of below 100%. For example Heim *et al* measured CerTN-L15 ΔR/R responses between 10 and 50% in cortical neurons electrically stimulated by 2 to 10 action potentials (ΔR/R measured with the dichroic method) [8]. However several recent studies have reported improved brightness and efficiency of fluorescent protein FRET constructs [45], [46] [47], [48]. In particular Mank et al developed a FRET probe that is more than twice as sensitive as CerTN-L15 (ΔR/R close to 150% *in vivo* in *Drosophila* motor neurons) [47]. Imaging these new probes with our technique will further improve sensitivity of calcium signal measurements.

In conclusion we have demonstrated that four-channel SU in two photon microscopy is beneficial for two main reasons: 1) it allows separations and quantification of spatially and spectrally overlapping fluorophores and 2) it increases the sensitivity to FRET based FCIP signals in depth in the brain.

## Supporting Information

Figure S1 Spectral sensitivity of the 4 detection channels used to unmix green1/green2 (A) or LuY/FITC in mixed solutions (B). Dichroic filters with cut-off wavelength at 510, 530 and 570 nm were used for both experiments. Laser excitation wavelengths were 850 nm and 860 nm for the green1/green2 and LuY/FITC experiments respectively.
(0.89 MB TIF)

Figure S2 Spectral sensitivity of the 4 channels used to unmix ECFP and EYFP. Dichroic filters with cut-off wavelength at 480, 510 and 550 nm were used.
(0.63 MB TIF)

Figure S3 SU of four fluorescent proteins. HEK cells co-transfected with YC3.1 and P2X2 were mixed with cells transfected with GFP or tdimer2 and platted onto glass cover slips. tdimer2 is a red fluorescent protein (FP) with a peak emission at 579 nm [49]. In the mixed cells sample, some contained ECFP and EYFP in the YC3.1 probe and were ATP-sensitive due to the presence of P2X2 channels, and some were either GFP or tdimer2 positive and not ATP sensitive. ECFP, GFP and EYFP emission spectra have significant overlap (A). tdimer2 is further apart but could still suffer from bleed-through from EYFP. As a consequence, it is not possible to separate these four FPs using conventional bandpass emission filters. Thanks to their large two-photon excitation spectra [30] all four FPs could be excited simultaneously with a single wavelength, although not with the same efficacy. The Ti:Sapphire laser wavelength was tuned to 800 nm. To demonstrate the ability to unmix four FPs with our 4-channels SU method, we imaged a region where the 3 types of cells were simultaneously present (B) and applied 50 mM ATP in perfusion (C). The contribution of each fluorophore was computed and a merged imaged obtained where ECFP, GFP, EYFP and tdimer2 were color-coded in blue, green, yellow, and red, respectively. (D) Plot of cell 1-3 contributions as a function of time. Some cells such as cell 1 were responding to ATP with an increase of EYFP and a simultaneous decrease of ECFP. These cells had very little GFP or tdimer2 contributions (less than 8% of the total signal). Some cells, like cells 2 and 3 in (B–C), were bright green (red) meaning that their contributions was purely GFP (tdimer) with the other fluorophores signals representing between 3 and 10% of the total signal (D2–3). tdimer2 measured in cell 3 decreases slowly with time probably due to photobleaching. This result demonstrates that we can separate the contributions of four FPs with significant spectral overlaps, in spite of the low spectral resolution of our system. Scale bar in C is 20 microns.
(1.64 MB TIF)

Figure S4 YC3.1 spectra in control condition was altered by the presence of a brain slice. Reference spectra were measured in 29 HEK cells transfected with YC3.1 without (solid line) and with (dashed line) a 300 micron thick rat neocortex brain slice between the cells and the objective. (mean+/−SEM)
(0.33 MB TIF)

Table S1 System specifications for each experiment. Optical elements from Chroma (*), Schott (‡), Olympus (§) or Omega (†)
(0.02 MB DOC)
