# Supplementary figures and images for: Spectral Unmixing: Analysis of Performance in the Olfactory Bulb In Vivo
Source: PLoS One. 2009 Feb 9;4(2):e4418. doi: 10.1371/journal.pone.0004418 (PMC2635473; doi:10.1371/journal.pone.0004418)

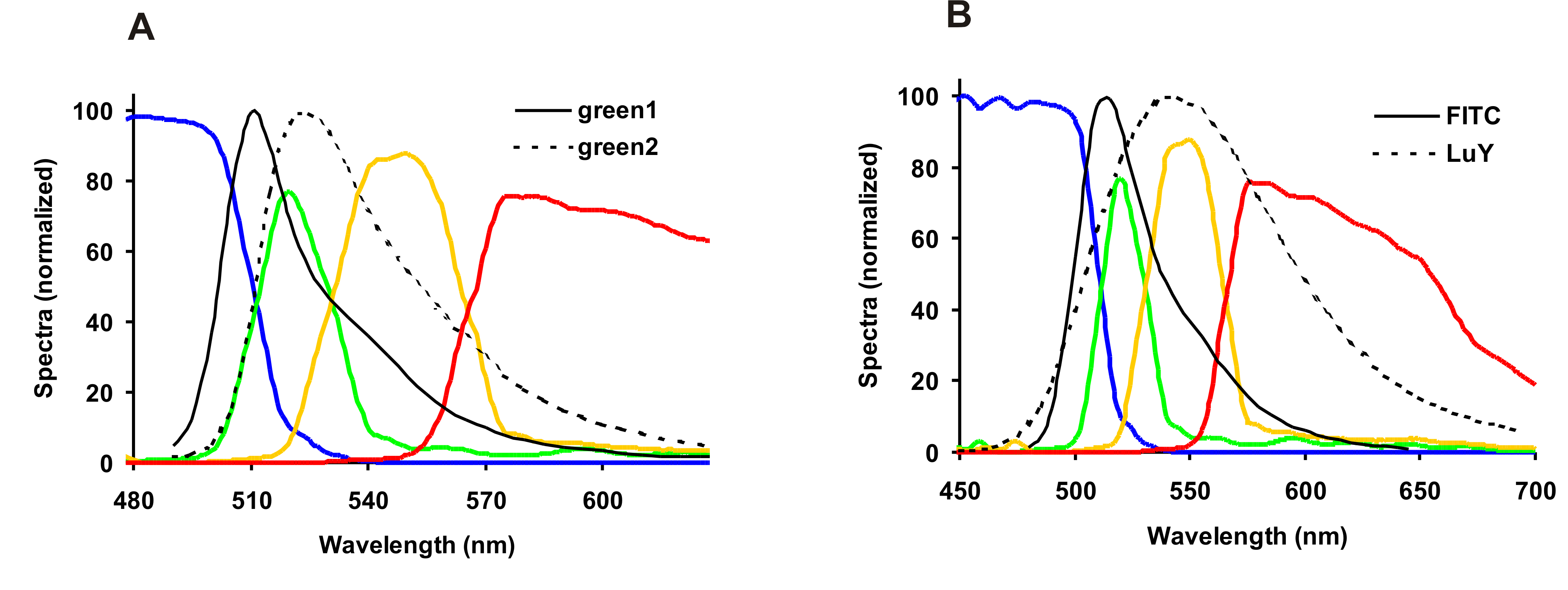

Supplement: Figure S1 — Spectral sensitivity of the 4 detection channels used to unmix green1/green2 (A) or LuY/FITC in mixed solutions (B). Dichroic filters with cut-off wavelength at 510, 530 and 570 nm were used for both experiments. Laser excitation wavelengths were 850 nm and 860 nm for the green1/green2 and LuY/FITC experiments respectively. (0.89 MB TIF) [file pone.0004418.s001.tif]

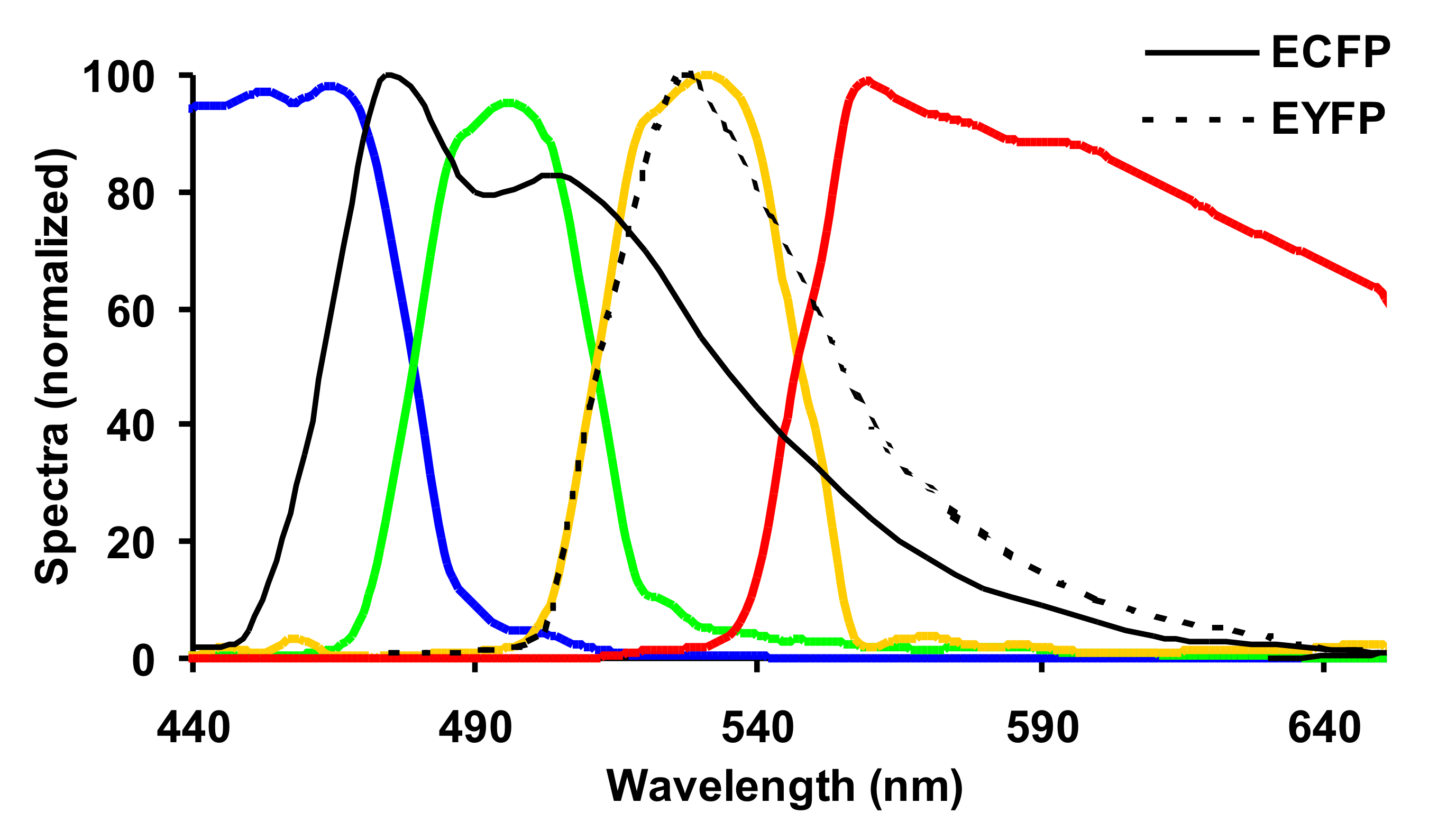

Supplement: Figure S2 — Spectral sensitivity of the 4 channels used to unmix ECFP and EYFP. Dichroic filters with cut-off wavelength at 480, 510 and 550 nm were used. (0.63 MB TIF) [file pone.0004418.s002.tif]

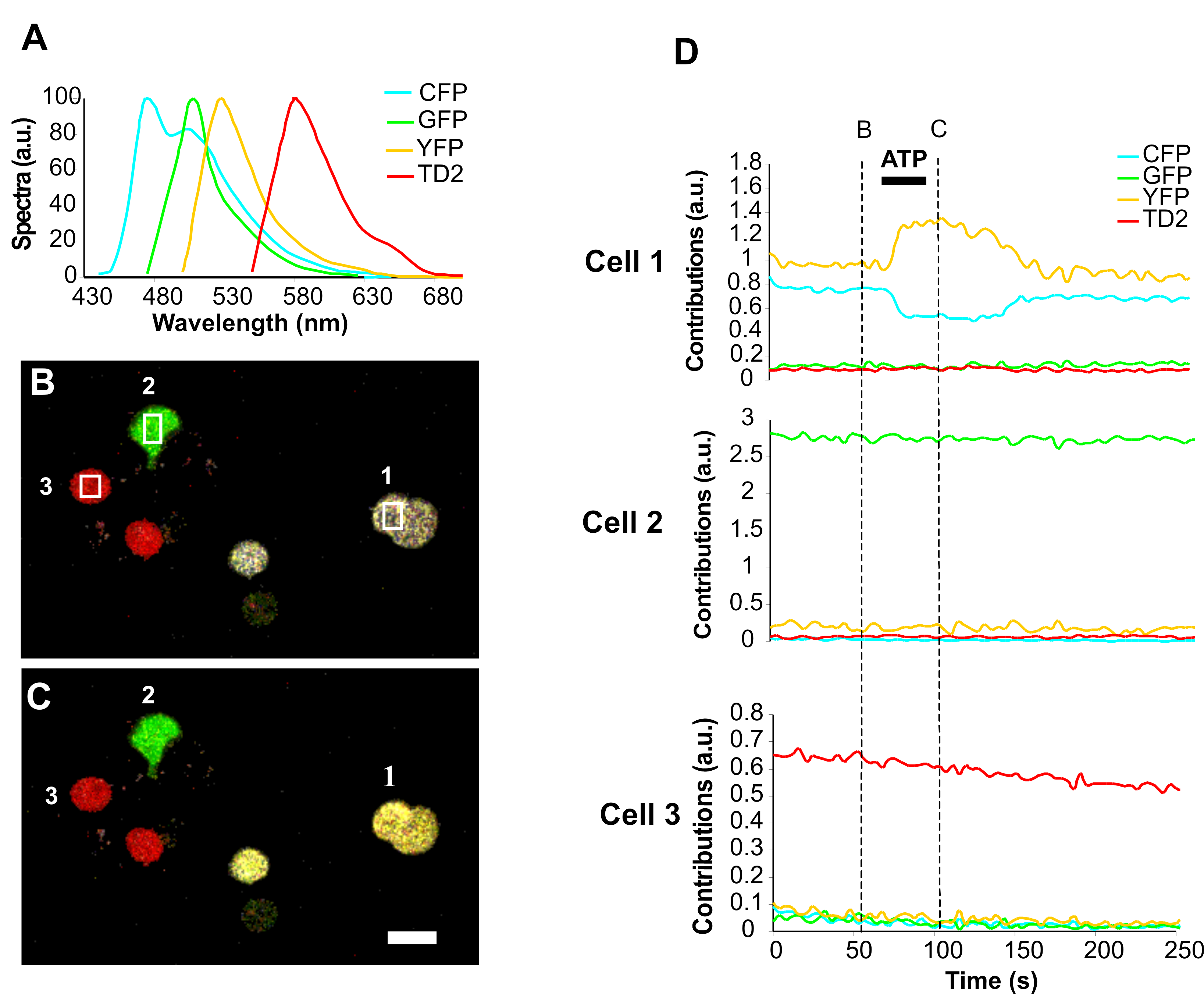

Supplement: Figure S3 — SU of four fluorescent proteins. HEK cells co-transfected with YC3.1 and P2X2 were mixed with cells transfected with GFP or tdimer2 and platted onto glass cover slips. tdimer2 is a red fluorescent protein (FP) with a peak emission at 579 nm [49]. In the mixed cells sample, some contained ECFP and EYFP in the YC3.1 probe and were ATP-sensitive due to the presence of P2X2 channels, and some were either GFP or tdimer2 positive and not ATP sensitive. ECFP, GFP and EYFP emission spectra have significant overlap (A). tdimer2 is further apart but could still suffer from bleed-through from EYFP. As a consequence, it is not possible to separate these four FPs using conventional bandpass emission filters. Thanks to their large two-photon excitation spectra [30] all four FPs could be excited simultaneously with a single wavelength, although not with the same efficacy. The Ti:Sapphire laser wavelength was tuned to 800 nm. To demonstrate the ability to unmix four FPs with our 4-channels SU method, we imaged a region where the 3 types of cells were simultaneously present (B) and applied 50 mM ATP in perfusion (C). The contribution of each fluorophore was computed and a merged imaged obtained where ECFP, GFP, EYFP and tdimer2 were color-coded in blue, green, yellow, and red, respectively. (D) Plot of cell 1-3 contributions as a function of time. Some cells such as cell 1 were responding to ATP with an increase of EYFP and a simultaneous decrease of ECFP. These cells had very little GFP or tdimer2 contributions (less than 8% of the total signal). Some cells, like cells 2 and 3 in (B–C), were bright green (red) meaning that their contributions was purely GFP (tdimer) with the other fluorophores signals representing between 3 and 10% of the total signal (D2–3). tdimer2 measured in cell 3 decreases slowly with time probably due to photobleaching. This result demonstrates that we can separate the contributions of four FPs with significant spectral overlaps, in spite of the low spectral [file pone.0004418.s003.tif]

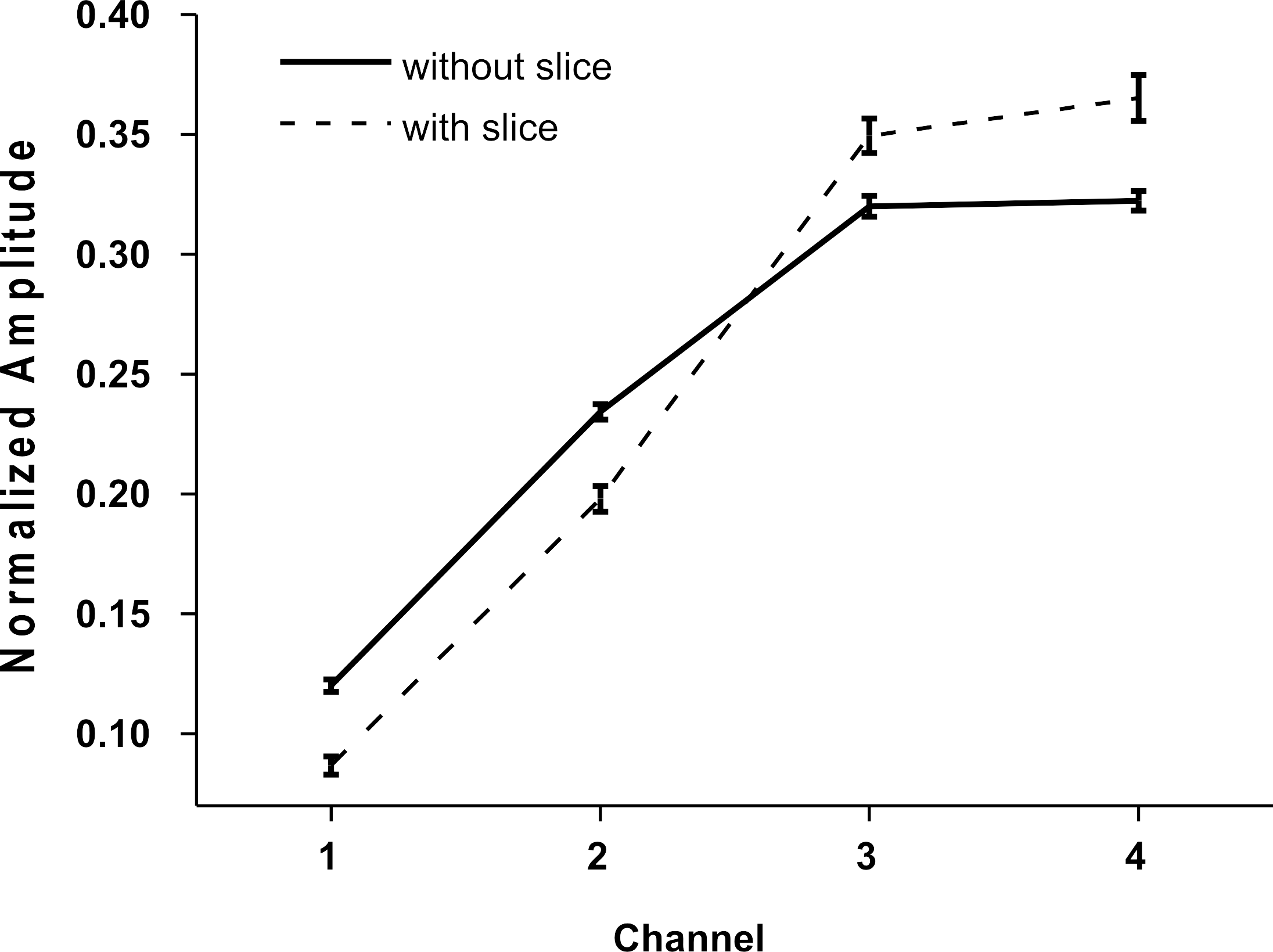

Supplement: Figure S4 — YC3.1 spectra in control condition was altered by the presence of a brain slice. Reference spectra were measured in 29 HEK cells transfected with YC3.1 without (solid line) and with (dashed line) a 300 micron thick rat neocortex brain slice between the cells and the objective. (mean+/−SEM) (0.33 MB TIF) [file pone.0004418.s004.tif]
