# Supplementary material for: Spectral Unmixing: Analysis of Performance in the Olfactory Bulb In Vivo
Source: PLoS One. 2009 Feb 9;4(2):e4418. doi: 10.1371/journal.pone.0004418 (PMC2635473; doi:10.1371/journal.pone.0004418)
Supplement: Table S1 — System specifications for each experiment. Optical elements from Chroma (*), Schott (‡), Olympus (§) or Omega (†) (0.02 MB DOC) [file pone.0004418.s005.doc]

**SUPPORTING TABLE 1**

| Fluorophores to be unmixed | Green1/Green2 or FITC/LuY | ECFP/EYFP,  ECFP/GFP/EYFP/tdimer2 | ECFP/EYFP bandpass (BP) method |
| --- | --- | --- | --- |
| DM2 | 510DCXR^*^ | 480DCXR^*^ | None |
| DM3 | 530DCXR^*^ | 510DCXR^*^ | 510DCXR^*^ |
| DM4 | 570DCXR^*^ | 550DCXR^*^ | None |
| Blue emission filter | Colored glass filter BGG22 2mm ^‡^ | Colored glass filter BGG22 2mm ^‡^ | None |
| Green emission filter | Low pass filter E700 ^*^ | Low pass filter E700SP2 ^*^ | Bandpass filter BP460-490 ^§^ |
| Yellow emission filter | Low pass filter E700SP2 ^*^ | Low pass filter E700SP2 ^*^ | None |
| Red emission filter | Low pass filter E700SP2 ^*^ | Low pass filter E700SP2 ^*^ | Bandpass filter 535DF35 ^†^ |

**Table S1: System specifications for each experiment.** Optical elements from Chroma (*), Schott (‡), Olympus (§) or Omega (†)
